# Whole Blood Transcriptome Characterization of 3xTg-AD Mouse and Its Modulation by Transcranial Direct Current Stimulation (tDCS)

**DOI:** 10.3390/ijms22147629

**Published:** 2021-07-16

**Authors:** Chiara Magri, Erika Vitali, Sara Cocco, Edoardo Giacopuzzi, Marco Rinaudo, Paolo Martini, Alessandro Barbon, Claudio Grassi, Massimo Gennarelli

**Affiliations:** 1 Department of Molecular and Translational Medicine, University of Brescia, 25123 Brescia, Italy; e.vitali002@unibs.it (E.V.); paolo.martini@unibs.it (P.M.); alessandro.barbon@unibs.it (A.B.); massimo.gennarelli@unibs.it (M.G.); 2 Genetics Unit, IRCCS Istituto Centro San Giovanni di Dio Fatebenefratelli, 25125 Brescia, Italy; edoardo.giacopuzzi@well.ox.ac.uk; 3 Department of Neuroscience, Università Cattolica del Sacro Cuore, 00168 Rome, Italy; sara.cocco@unicatt.it (S.C.); marco.rinaudo@unicatt.it (M.R.); claudio.grassi@unicatt.it (C.G.); 4 Wellcome Centre for Human Genetics, Oxford University, Oxford OX3 7BN, UK; 5 NIHR Biomedical Research Centre, Oxford OX3 7BN, UK; 6 Fondazione Policlinico Universitario A. Gemelli IRCCS, 00168 Rome, Italy

**Keywords:** 3xTg-AD mouse, RNA-Seq, Alzheimer’s disease, transcranial direct current stimulation

## Abstract

The 3xTg-AD mouse is a widely used model in the study of Alzheimer’s Disease (AD). It has been extensively characterized from both the anatomical and behavioral point of view, but poorly studied at the transcriptomic level. For the first time, we characterize the whole blood transcriptome of the 3xTg-AD mouse at three and six months of age and evaluate how its gene expression is modulated by transcranial direct current stimulation (tDCS). RNA-seq analysis revealed 183 differentially expressed genes (DEGs) that represent a direct signature of the genetic background of the mouse. Moreover, in the 6-month-old 3xTg-AD mice, we observed a high number of DEGs that could represent good peripheral biomarkers of AD symptomatology onset. Finally, tDCS was associated with gene expression changes in the 3xTg-AD, but not in the control mice. In conclusion, this study provides an in-depth molecular characterization of the 3xTg-AD mouse and suggests that blood gene expression can be used to identify new biomarkers of AD progression and treatment effects.

## 1. Introduction

Alzheimer’s Disease (AD) is a chronic and progressive neurodegenerative disorder and, according to the World Health Organization, the first cause of dementia worldwide [1]. The main anatomical features of AD are the presence of cerebral extracellular beta amyloid plaques and neurofibrillary tangles. In addition, AD is characterized by brain atrophy, reduction of brain plasticity and alteration of functional connectivity [2,3,4,5].

Due to the increase in global population and life expectancy, AD can be considered a global issue, and the development of therapies an emergency; indeed, it is estimated that only in the United States the number of people with AD will triplicate by 2050 [6]. However, to date the only available treatments aim to reduce the symptoms of the disease and to slow down its progression; therapies that stop or reverse AD evolution have not been identified yet and several trials performed in the last years have failed [7,8].

Among the several animal models generated to better understand the pathological mechanisms of AD, the triple transgenic (3xTg-AD) mouse is one of the most widely used, since it allows mimicking the progression of the human disease [9,10]. 3xTg-AD mice are characterized by the presence of three mutations associated with familiar AD: the human APP Swedish, PSEN1 M146V and MAPT P301L mutations. The translation of these overexpressed transgenes appears restricted to the central nervous system of 3xTg-AD mice and it induces the development of both plaques and tangles, allowing the study of these pathological events in a single model. The beta amyloid plaques appear in the hippocampus and cortex of 3xTg-AD mice at six months of age, whereas neurofibrillary tangles appear in the hippocampus only at twelve months [11,12]. Starting from four months of age, 3xTg-AD mice show recognition and exploratory memory impairments, with alterations in locomotor activity [10]; deficits in synaptic plasticity and a decreased Long-Term Potentiation (LTP) appear at six months of age, before the onset of plaques and tangles [11], and at this age 3xTg-AD mice also develop neuroinflammation, that gets worse with aging [12]. Despite that the anatomical and behavioral features of 3xTg-AD mice have been well characterized, the transcriptomic profile of this model remains poorly investigated. A few studies performed on 3xTg-AD mice hippocampi and prefrontal cortices highlighted altered expression profiles of genes involved in calcium homeostasis, mitochondrial functioning and inflammatory response, and of genes known to be involved in AD [13,14]. To date, only one study has characterized the whole blood transcriptome profile of the 3xTg-AD providing important information about gene expression in the 3xTg-AD mice at three and twelve months of age and about the gene expression correlation between blood and the hippocampus [15].

**Figure 1:**
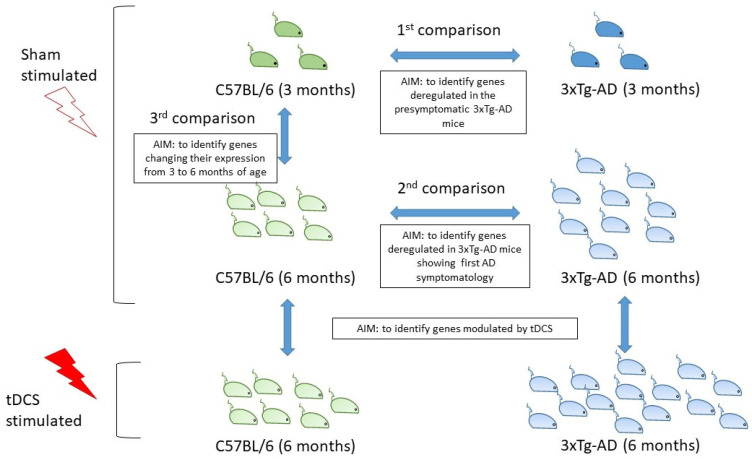
Study design.

As reported in Figure 1, the main aim of this study is to further characterize the blood transcriptome profile of the 3xTg-AD mouse by analyzing mice at three and six months of age, in order to identify peripheral biomarkers of AD progression and prognosis. In particular, we studied the expression profile of pre-symptomatic three-month-old mice and of symptomatic six-month-old mice compared to age-matched control mice (C57BL/6); in this way, we could better analyze which genes and pathways are modulated by the 3xTg-AD mice’s genetic background and which are modulated by age and the onset of AD symptomatology. To assess if gene expression changes in blood can also be exploited to study the brain response to external stimulation, we also analyzed the transcriptome profile of mice that underwent anodal transcranial Direct Current Stimulation (tDCS). tDCS is a noninvasive brain stimulation technique that is able to modulate cortical excitability through the release of a weak electrical current. In particular, anodal stimulation is known to depolarize neurons, to increase the probability of action potentials occurring and to induce synaptic plasticity [16,17,18]. The effects of tDCS on brain gene expression have been previously assessed in different animal models, revealing its ability to modulate the expression of genes involved in the mechanisms of synaptic plasticity and neuronal activity [19,20,21,22]; however, to date no studies have investigated its effects at the blood level.

## 2. Results

Globally, the blood transcriptome of 42 mice was sequenced. The spike-in analysis using sequins synthetic RNA revealed a gene detection sensitivity of 0.059 (attomol/uL). The correlation between the input concentration and the measured concentration of the sequins was 0.90 ± 0.04 and the r^2^ of the linear model was 0.81 ± 0.07 (Supplementary Figure S1). The high correlation indicates excellent recovery of the spike-in mix during the entire library’s preparation, high technical reproducibility and reduced/absent technical bias due to library preparation and sequencing workflow.

After sequencing and quality checks, we generated blood transcriptome profiles for 15,613 genes from 42 mice, stratified in six different groups (Table 1).

**Figure 2:**
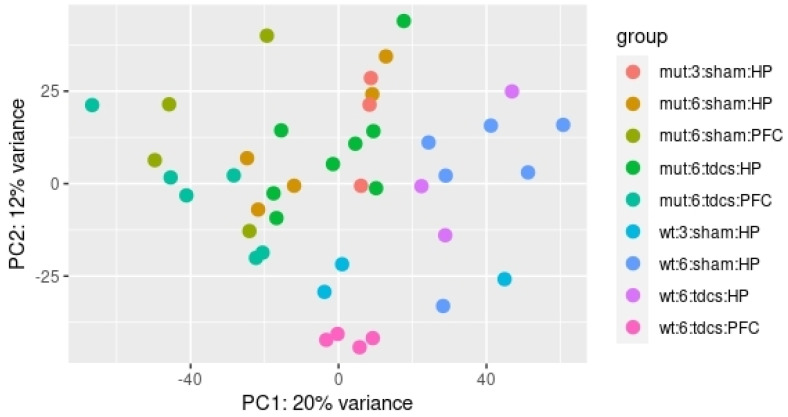
Scatterplot of the Principal Component Analysis.

Principal Component (PC) analysis revealed that the first five principal components account for more than 51% of phenotype variability observed among the mice (Supplementary Figure S2). In particular, the first PC explains 20% of the whole blood transcriptomic variability (Figure 2) and it was strongly correlated with the “mouse strain” (r^2^ = −0.64) and “site of stimulation” (r^2^ = −0.63) variables.

### 2.1. Differential Expression Analyses

In order to characterize the whole blood expression profile of the 3xTg-AD mouse strain, we analyzed three different comparisons as described below. The top ten differentially expressed genes (DEGs) from each comparison are reported in Table 2, while the summary statistics of all analyzed genes are reported in Supplementary Table S1.

1. **3-month-old (3 mo.) 3xTg-AD vs. 3 mo. C57BL/6 mice**. Of the 15,613 genes studied, 69 were not analyzed in this comparison because of a total read count equal to zero. This analysis identified 470 genes (193 up- and 277 down-regulated) deregulated in 3xTg-AD mice at the pre-symptomatic stage, mostly representing the effect of the specific genetic background of this strain. The DEG with the highest log_2_ fold change was the neuropeptide Y gene (*Npy*). This gene, which is usually poorly expressed in whole blood [23,24], was not detected in the 3 mo. C57BL/6 (WT mice), while it was expressed at very high levels in 3 mo. 3xTg-AD mice (normalized mean number of reads: 6295 ± 2565). Among the top ten DEGs, *Npy*, *Serpine2* and *Acp1* genes were previously found associated at different levels with AD [25,26,27,28,29,30]. DEGs in 3 mo. 3xTg-AD mice were significantly enriched (FDR < 0.05) for genes belonging to the biological processes of leukocyte chemotaxis, blood coagulation, response to lipopolysaccharide, cellular metal ion homeostasis, positive regulation of hemopoiesis, regulation of cell-cell adhesion, negative regulation of secretion by cell, and regulation of blood coagulation (Supplementary Table S2).
2. **6-month-old (6 mo.) 3xTg-AD vs. 6 mo. C57BL/6 mice**. Of the 15,613 genes selected, only one was not analyzed in this comparison because of a total read count equal to zero. This comparison identified 1551 DEGs (912 up- and 639 down-regulated) deregulated in 3xTg-AD mice at a stage where they show the first symptoms of AD pathology. Thus, observed differences in gene expression can be either a direct consequence of the genetic background of this strain, or a secondary effect of the appearance of first AD symptoms. Among the top ten DEGs, those with the highest log_2_ fold change is the kallikrein 1-related peptidase b22 (*Klk1b22*). The identified DEGs were significantly enriched for genes belonging to the REACTOME pathway of immunoregulatory interactions between a lymphoid and a non-lymphoid cell (Supplementary Table S2).
3. **6 mo. C57BL/6 vs. 3 mo. C57BL/6 mice**. Of the 15,613 genes selected, 18 were not analyzed in this comparison because of a total read count equal to zero. This comparison identified 271 DEGs (214 up- and 57 down-regulated) changing their expression from 3 to 6 months of age. Among DEGs, the expression of *Ddit4* and *Folr1* has been previously shown to be age-related and associated with AD [31,32,33,34]. Pathway enrichment analysis revealed a significant enrichment for genes belonging to the keratinocyte differentiation pathway (Supplementary Table S2).

The Alzheimer KEGG Pathway (entry: mmu05010) has also been tested. A total of 303 out of the 368 genes included in this pathway were also analyzed in this study (Supplementary Table S1). DEGs derived from the three aforementioned comparisons were not significantly enriched of genes belonging to this pathway. However, when the analysis was extended to all genes with FDR < 0.20, we observed a significant enrichment of genes of the Alzheimer KEGG Pathway among DEGs of the second comparison. Indeed, this group of genes showed 1.22 more genes of the Alzheimer KEGG pathway than expected by chance (hypergeometric test *p* = 0.026).

### 2.2. Venn Diagram Analysis

**Figure 3:**
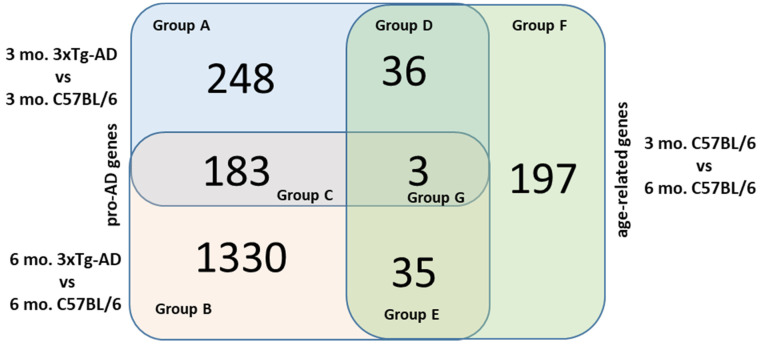
Venn diagram of the three comparisons performed among sham mice.

In order to obtain further insights into the biological mechanisms underlying the observed gene expression modulation, the list of DEGs obtained from the three aforementioned comparisons were intersected via Venn diagram (Figure 3).

Thus, we obtained a group derived by the intersection of all three comparisons that includes three genes (*Upb1*, *Chil3* and *Farsa*) and three groups derived by the pair-wise intersections, named Group C, Group D and Group E and described below.

#### 2.2.1. Group C

**Figure 4:**
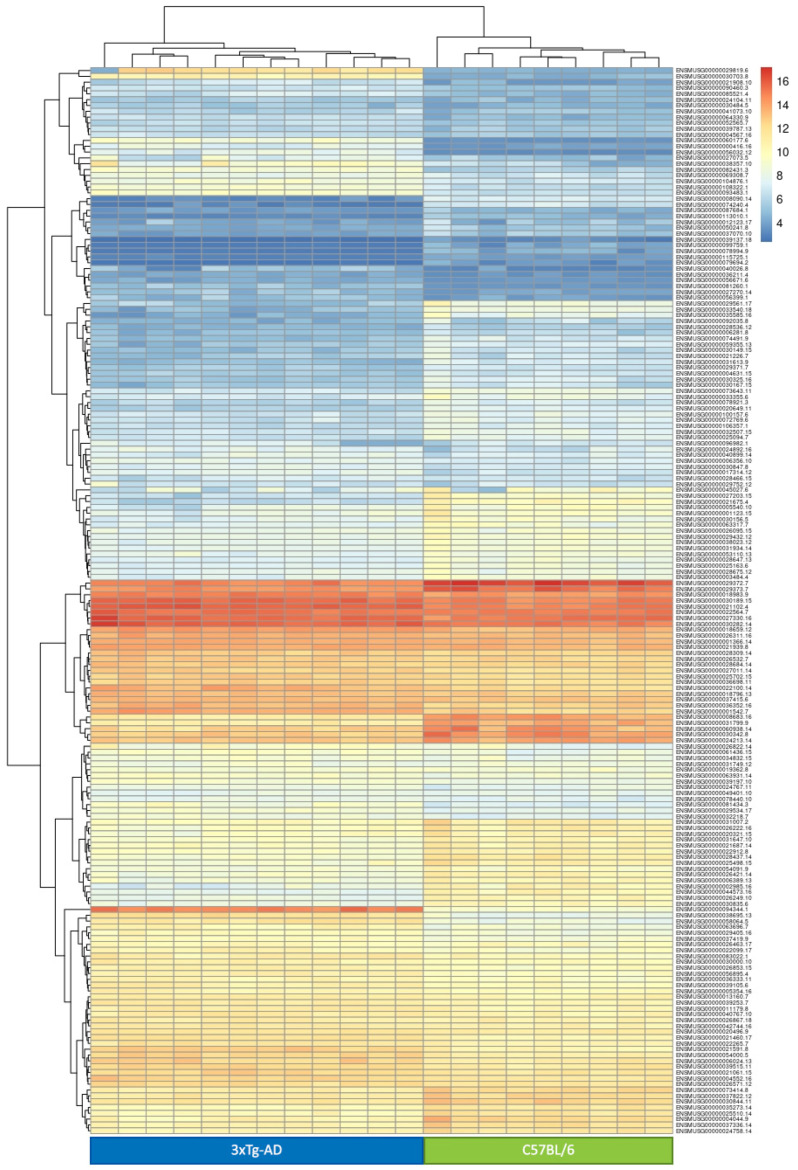
Heatmap of the DEGs in group C.

In this group, there are genes that are deregulated both in the first and second comparisons and likely represent genes that are constitutively deregulated in the 3xTg-AD strain compared to the WT one, independently of age and presence of the AD symptomatology. Thus, we can assume that gene expression differences seen for these genes are directly influenced by the different genetic background of the strains analyzed. In this group, we observed 183 genes (Figure 3), a number 3.99-fold higher than expected by chance (randomized test *p* < 1 × 10^−5^). The list of these genes is reported in Supplementary Table S1. Intriguingly, we noted that the log_2_ fold-change values resulting from the two comparisons for these genes are strongly positively correlated (r^2^ = 0.92), implying that gene expression is similarly modulated in 3 mo. 3xTg-AD mice and 6 mo. 3xTg-AD mice, compared to WT mice of the same age. As depicted in the Heatmap (Figure 4), among these genes, some were expressed at a moderate/high level in the 3xTg-AD mice, and not expressed, or expressed at a very low level, in WT mice.

The Over-Representation Analysis (ORA) revealed an enrichment of genes belonging to the biological processes of blood coagulation and regulation of blood coagulation (Table 3).

#### 2.2.2. Group D

The intersection of DEGs from the first and third comparisons represents genes whose expression changes from 3 to 6 months of age in the WT mice and also differentiate the AD mouse model from WT at the pre-symptomatic stage. In this group, we found 36 genes (Figure 3), a number 4.9 times higher than expected by chance (randomized test *p* < 1 × 10^−5^). The list of these genes is reported in Supplementary Table S1. We observed a positive correlation (r^2^ = 0.98) between the fold-change value of DEGs from the first and third comparisons. In other words, for these genes, the expression profiles of pre-symptomatic 3xTg-AD mice are more similar to that of 6 mo. WT mice than to that of WT mice of the same age. This result is in line with the hypothesis of Gatta and collaborators [14] that the genetic background of the 3xTg-AD mice could induce a biological alteration resulting in premature aging. We did not observe any significant enrichment considering all genes in this group, while we observed a significant enrichment for the pathway of response to cAMP when considering only down-regulated genes (Table 3).

#### 2.2.3. Group E

This group includes genes whose expression changes with age in the WT mice and which are differentially expressed in the symptomatic 6 mo. 3xTg-AD mice. In this group, we found 35 genes (Figure 3), a number slightly higher (1.3) than expected by chance (randomized test *p* = 0.045). The list of these genes is reported in Supplementary Table S1. The analysis revealed a negative correlation (r^2^ = −0.94) between the effect size of DEGs in the second and third comparisons; that is, the gene expression perturbation observed in 6 mo. 3xTg-AD mice for these genes is opposite to that induced by age. We did not find these genes significantly enriched for any pathways when stratified in either up- or down-regulated genes.

#### 2.2.4. Groups A, B and F

From the intersection analysis, we identified three groups, namely A, B and F, that correspond to genes that were deregulated exclusively in one of the three aforementioned comparisons. Group A includes genes that were DEGs uniquely in the first comparison; Groups B and F includes those that were unique DEGs in the second and third comparisons, respectively (Figure 3, Supplementary Table S1).

Group A includes 248 genes differentially expressed only in the pre-symptomatic 3xTg-AD mice. Down-regulated genes of this group resulted in being enriched with genes belonging to the Reactome pathway of Signaling by Interleukins (Table 3).

Group B includes 1330 genes differentially expressed only in the 6 mo. 3xTg-AD mice showing the first symptoms of AD pathology. Up-regulated genes of this group were mainly enriched with genes belonging to biological processes of selective autophagy and the negative regulation of peptidase activity (Table 3). Since, compared to DEGs identified in the second comparison, this group includes only genes deregulated in the symptomatic 6 mo. 3xTg-AD mice, we could hypothesize that the aforementioned pathways are those mainly modulated by the onset of the first symptoms of the AD pathology.

Group F includes 197 genes differentially expressed only in the 6 mo. WT mice compared to the 3 mo. WT mice. This group is mainly enriched with genes belonging to the biological processes of keratinocyte differentiation, actin cytoskeleton organization, multicellular organismal water homeostasis, intermediate filament cytoskeleton organization and to Reactome pathway of Phase I—Functionalization of compounds (Table 3).

### 2.3. tDCS Effects on Whole Blood Transcriptome

In order to study the effects of tDCS on whole blood transcriptome, we compared the gene expression of the 6 mo. mice undergoing sham stimulation to that of mice undergoing tDCS. This analysis was performed in the two strains separately. A total of 25 DEGs were identified in the comparison involving the 3xTg-AD mice, whereas no DEGs were observed in the same comparison performed in C57BL/6 mice (Supplementary Table S1). The list of these genes is reported in Table 4.

**Figure 5:**
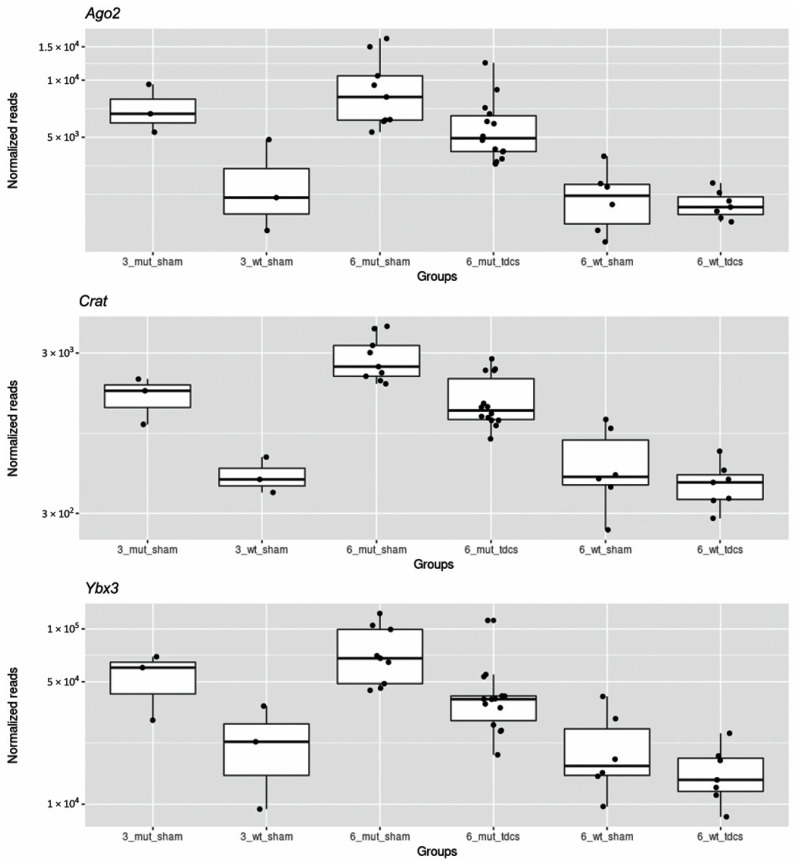
Boxplot of normalized reads for genes modulated by tDCS and deregulated in the 3xTg-AD mouse. Normalized reads were those calculated and used by DESeq2 software. A log scale is used for the Y axis. It is of note that 6 mo. 3xTg-AD mice undergoing tDCS stimulation (6_mut_tdcs) have expression levels that are in between that of 6 mo. 3xtg-AD mice undergoing sham stimulation (6_mut_sham) and 6 mo. WT mice undergoing sham stimulation (6_wt_sham).

Four of these DEGs (*Ap1b1*, *Ogdh*, *Rnf123* and *Mical3*) were also present in Group B, and three (*Crat*, *Ybx3*, *Ago2*) were included in Group C. For these three genes, Boxplots (Figure 5) revealed that mice stimulated with tDCS show a level of expression that approximate to that of WT sham mice. No gene groups resulted in being significantly enriched from gene-set enrichment analysis.

## 3. Discussion

In this study, we characterized the whole blood transcriptomic profile of the 3xTg-AD mouse strain, highlighting two main findings.

First, we identified 183 genes that were significantly deregulated in the 3xTg-AD mice compared to the WT mice independently of age (Group C), and that could represent a direct signature of the different genetic background of the two strains studied (3xTg-AD and C56BL/6). Intriguingly, among these genes, some were expressed at a very high level in the 3xTg-AD mice, and not expressed, or expressed at a very low level, in WT mice. The two most deregulated genes among these were *Npy,* which encodes for the neuropeptide y, a polypeptide with an important neuroprotective and anti-neurodegenerative role [35], and *Gdpd3*, which encodes for lysophospholipase D. *Npy* has an essential role in neuroprotection against toxic stimuli [36], in reduction of inflammation [37] and in mechanisms of learning and memory [38]. Moreover, the role of *Npy* in neurodegenerative diseases has been largely investigated [30]. In different AD mouse models, including the 3xTg-AD, the expression of *Npy* seemed to be reduced [39,40,41]. However, studies in other models showed an increase of NPY protein levels in specific brain regions [42,43]. Despite these contrasting results, which could depend on the peculiarities of each model, there is evidence of a reduction in NPY protein levels in the brains of AD patients [44], and evidence of a reduction, or no alteration, of the protein level in plasma [45,46]. These results suggest that *Npy* could have an important role in AD and it could be considered as a possible therapeutic target for AD and other neurodegenerative diseases [29]. Concerning the *Gdpd3* gene, its higher expression in 3xTg-AD mice compared to WT mice is intriguing in light of the fact that the human ortholog maps in the 16p11.2. Micro duplication of this locus has been associated with developmental delay, intellectual disability, behavioral problems, autism, schizophrenia, bipolar disorder and with psychosis in Alzheimer’s disease [47]. Among the genes of Group C, we also observed a down-regulation of *Apoe*. A reduced expression of this gene in the blood of 3xTg-AD mice was already reported [15] and reflects what was observed in AD patients [48,49,50], which was also correlated to a decrease in hippocampal volume [51] and an increase in the amount of cerebral beta amyloid [50,52]. Our results confirm the role of *Apoe* as a peripheral biomarker for AD. Further, our analyses of Group C genes revealed an enrichment of genes associated with blood coagulation and its regulation. The alteration of coagulation mechanisms in AD has been previously investigated; different studies observed increased levels of thrombin [53] and fibrinogen [54] in the brains of AD patients and an alteration in the levels of different hypercoagulation markers in the peripheral blood of AD patients [55]. Concerning mouse models, a study focused on the activity of platelets in 3xTg-AD mice showed an increased tendency of platelets to adhere and to form clotting [56], whereas the APP23 mice showed a pre-activated state and an accelerated activation of platelets [57]. Despite this evidence of an enhanced coagulation mechanism in AD, we also observed a down-regulation of genes involved in the activation of coagulation. This last result is in accordance with a previous study that reported an impairment of clotting formation and a reduction in clotting formation time, both in AD patients and in Tg6799 AD mice model [58].

The second most relevant finding of this study were the 1330 DEGs (Group B) differentially expressed only in the 6 mo. 3xTg-AD mice; that is, they were neither age-related genes nor deregulated in the pre-symptomatic 3xTg-AD mice. At six months of age, the 3xTg-AD mice begin to show the main features of the disease, such as the presence of beta amyloid plaque formation both in cortex and hippocampus [11], tau hyperphosphorylations, altered microglia activation, neuroinflammation [12], alterations in basal synaptic transmission and in LTP, learning impairment and cognitive decline [11,12]. Since the genes of Group B were deregulated only in symptomatic 6 mo. 3xTg-AD mice, we could speculate that their expression is modulated by the onset of the pathological phenotype of AD, which begins to appear in mice’s brains at only six months of age. For this reason, these genes could be considered as potential peripheral biomarkers of the first symptomatology onset of the pathology; they could be useful to evaluate the efficacy of potential pharmacological and non-pharmacological therapies.

Group B did not result in being enriched with genes belonging to a specific pathway; however, up-regulated genes were enriched with genes of the selective autophagy process and the negative regulation of peptidase activity process. Autophagy is a cell process mediated by lysosomes responsible for the degradation of misfolded protein aggregates and damaged organelles. Recent studies have suggested that selective autophagy is closely implicated in neurological diseases [59]. Indeed, in many neurological disorders, the failure of the selective autophagy processes determines abnormal protein aggregation that causes irreversible damages, especially in the brain [60,61].

To complete the analysis and evaluate how the expression profile changes during the mouse physiological growth, we compared C57BL/6 mice at three and six months of age (third comparison), and we identified 271 DEGs. Among these, 36 genes (Group D) resulted in being differentially expressed also in the comparison of 3 mo. 3xTg-AD vs. 3 mo. C57BL/6 mice, and their expression changes were concordant, i.e., all up-regulated genes in 3 mo. 3xTg-AD were also up-regulated in 6 mo. C57BL/6 mice (and down-regulated genes as well). These observations suggest that, in the blood of the 3 mo. 3xTg-AD mice, there are genes whose expression is more similar to that of a 6 mo. mouse rather than that of a 3 mo. one. Our findings are in line with those presented by Gatta et al., where the authors proposed a set of genes whose expression is modulated by age as a major contributing factor in AD [14]. Indeed, Gatta et al. have previously suggested an alteration in the expression of genes related to aging in 3 mo. 3xTg-AD mice; in particular, they observed an altered expression of genes related to mitochondrial functioning, inflammatory response and calcium homeostasis, in neuronal proliferation, synaptic plasticity and neuronal survival. Among age-related genes that are down-regulated in 3 mo. 3xTg-AD mice, we observed an enrichment of genes involved in the response to the AMP cyclical (cAMP) pathway. In particular, all of the four genes in this pathway were related to apoptosis and cell proliferation: three genes (*Fos*, *Fosb*, *Junb*) encode for subunits of the transcription factor complex AP-1, important regulators of cell proliferation, differentiation, transformation and apoptosis; the fourth, *Dusp1*, whose expression is controlled by the AP-1 complex, is a negative regulator of cell proliferation. Cell proliferation, apoptosis and senescence, fundamental for the maintenance of brain tissue homeostasis, are altered in the brains of AD patients, and they are supposed to be involved in the development and progression of the disease [62,63,64]. Despite neurons being known to be typically in a quiescent state in the adult nervous system, cell cycle reactivation was observed in neurodegenerative diseases [64,65] as a consequence of mitotic stimuli, such as the presence of beta amyloid plaques and tau tangles [66]. Despite the expression of proteins involved in the cell cycle, this cannot be typically concluded by neurons and it leads to neuronal apoptosis [67], another mechanism impaired in AD [68,69]. In accordance with our results, the overexpression of several cell cycle proteins was also observed in peripheral lymphocytes of AD patients [70].

No firm conclusion can be drawn from the deregulation of age-related genes in 6 mo. 3xTg-AD mice. Indeed, although we observed some age-related genes differentially expressed in the 6 mo. 3xTg-AD mice, their number was only slightly higher than expected and no enriched pathways were observed.

Ochi and colleagues have recently reported the characterization of blood and hippocampal transcriptome of the 3xTg-AD mouse by microarray analysis [15], through which they identified 379 genes that were differentially expressed in the blood of 3xTg-AD mice. A total of 285 genes were also analyzed in our study and, despite the methodological differences between the two studies, we observed a significant (hypergeometric *p* = 0.0014) enrichment (1.52 fold) of them among genes differentially expressed in the 3xTg-AD mice (DEGs of the first and second comparison). These results corroborate the involvement of these genes in the clinical phenotype of the mouse (Supplementary Table S1).

As a proof of the principle that brain stimulations have an effect on the brain with consequences that can also be observed at the peripheral level, we studied the blood transcriptome profile of mice that underwent anodal tDCS. tDCS has been largely studied for its effects on synaptic plasticity and neuronal excitability [16,17], and different studies in animal models have shown its effects on brain gene expression. In rat cerebral cortices, the administration of tDCS was observed to modulate the expression of several genes involved in synaptic plasticity, such as neurotransmitter receptors [22], plasticity related genes [71], and genes coding for the major histocompatibility complex I [21]. In addition, a study on C57BL/6 mice revealed that tDCS acts on synaptic plasticity through the modulation of *Bdnf* expression [19]. Despite the evidence that, in the brain, tDCS can modulate the expression of genes related to synaptic plasticity, until now no studies have investigated its effects on blood transcriptome. This information, however, could be very useful in order to identify peripheral biomarkers of synaptic plasticity that could be applied in diagnosis and prognosis of AD.

Our study identified 25 genes whose expression was modulated by tDCS in the 3xTg-AD mice. According to the Genome Expression Database (GXD) [72], 21 of these genes are expressed both in mice hippocampi and prefrontal cortices, sites of stimulation in our model. This result suggests that tDCS is able to modulate, directly or indirectly, the gene expression of peripheral tissues, such as blood, and it suggests that blood gene expression profiles could be used as biomarkers of synaptic plasticity.

Considering the evidence about the utility of tDCS in the treatment of neurodegenerative diseases, including AD [73], we investigated if the stimulation could restore the expression of some genes that were impaired in 3xTg-AD mice. Among the 25 genes whose expression was modulated by tDCS, three (*Crat*, *Ybx3* and *Ago2)* were deregulated in the 3xTg-AD mice independently of age. For all these genes, 3xTg-AD mice that underwent tDCS showed expression levels comparable to those of WT mice and significantly lower than 3xTg-AD undergoing sham stimulation. This result suggests that tDCS probably acts by normalizing the activity of some biological pathways that are altered in the pathology.

No genes were found deregulated in C57BL/6 mice undergoing tDCS. This negative result finds a possible explanation in the fact that the mice used for this comparison were fewer than the 3xTg-AD mice and therefore the analysis had a lower statistical power. However, another possible explanation could be found in the fact that tDCS restores mainly the expression of genes impaired in 3xTg-AD mice, and if these pathways are not impaired, as in the WT model, its effect is minimal and not detectable when analyzing few animals.

Our study depicts the whole blood transcriptomic profile of 3xTg-AD mice at three and six months of age by comparing their transcriptomic profiles with those of WT C57BL/6 mice of the same ages. When interpreting the results, we should keep in mind that the genetic backgrounds of these strains are similar, but not identical, since 3xTg-AD mice have a hybrid C57BL/6;129 genetic background. For this reason, we cannot completely rule out that some differences observed among the groups could be due to the different genetic background rather than to transgenic mutations of 3xTg-AD mice. Despite this limitation, the comparison of 3xTg-AD and C57BL/6 mice remains important in light of the fact that the C57BL/6 genome is the mouse reference genome, a lot of expression data are available for this strain, and it has previously been used as a control strain for 3xTg-AD mice [74,75,76].

Mice used in this study to characterize the 3xTg-AD strain underwent a sham stimulation. Although previous studies confirmed that the sham stimulation has no impact on the samples’ corticospinal excitability [77], we could not completely rule out that it could have any effect on peripheral gene expression. In any case, the eventual effect induced by the sham stimulation would be compensated by the fact that both strains underwent the same sham stimulation.

In conclusion, this study allowed a better characterization of the 3xTg-AD mouse strain from a molecular point of view. As expected, we found a modulation of pathways previously known to be altered in AD. In addition, this study highlighted that events that affect synaptic plasticity, such as the onset of the neurodegenerative diseases or brain stimulation techniques, such as tDCS, affect the expression profiles of the peripheral tissues. This last result suggests that peripheral gene expression can be used as a biomarker to study the progression of the pathology and the effects of possible pharmacological or non-pharmacological therapies.

## 4. Materials and Methods

### 4.1. Animal Models

Twenty-six male triple transgenic AD (3xTg-AD) mice, harboring human APP Swedish, PSEN1 M146V and MAPT P301L mutations, and sixteen C57BL/6 wild-type (WT) mice were analyzed in this study (Table 1). The 3xTg-AD and C57BL/6 mouse colonies were established and maintained at the Animal Facility of the Università Cattolica from breeding pairs (Jackson Laboratory). The animals were housed under a 12-h light-dark cycle at a temperature of 22–23 °C and a humidity of 60–75%.

### 4.2. tDCS Protocol

For tDCS stimulation, we adopted a unilateral epicranial electrode arrangement [19,78,79]. Specifically, the active electrode was composed of a tubular plastic cannula with an internal diameter of 3.0 mm, filled with saline solution (0.9% NaCl) just prior to tDCS; the counter electrode consisted of a rubber-plate electrode enclosed in a wet sponge (5.2 cm^2^). The center of the active electrode was positioned 1 mm posterior and 1 mm lateral to the bregma in mice subjected to hippocampal stimulation, and 1.7 mm anterior and 0 mm lateral to the bregma in mice which underwent prefrontal cortex stimulation [80]. The counter electrode was placed over the ventral thorax. The implant of the epicranial electrode was made under anesthesia by an intraperitoneal injection of a cocktail with ketamine (87.5 mg/Kg) and xylazine (12.5 mg/Kg). During surgery, the temperature was maintained at 37 °C. The scalp and underlying tissues were eliminated, and the electrode was positioned on the skull using a carboxylate cement (3M ESPE, Durelon, 3M Deutschland GmbH, Germany). After surgery, mice were placed in individual cages and were allowed to recover for 3–5 days before undergoing tDCS. For hippocampal stimulation, tDCS protocol consisted of 3 single stimulations (current intensity of 250 μA for 20 min, current density of 35.4 A/m^2^) delivered on 3 consecutive days, once per day. For prefrontal cortex stimulation, tDCS protocol consisted of 6 single stimulations (current intensity of 250 μA for 15 min, current density of 35.4 A/m^2^) delivered over 2 weeks, for 3 consecutive days per week. We adopted the “anodal” tDCS configuration, corresponding to a positive electric field (positive electrode over the hippocampus or the prefrontal cortex). A battery-driven constant current stimulator (BrainSTIM, EMS, Italy) was used to deliver tDCS. The current intensity was ramped for 10 s to prevent a stimulation break effect. tDCS was applied to awake mice at approximately the same time of day (around 10 a.m.). The animals were observed during tDCS and no abnormal behaviors were detected related to the stimulation. In brain tissues obtained from mice subjected to tDCS, no morphological alterations were found. Control mice received a sham stimulation (the same manipulation as in tDCS protocol was performed, but no current was applied). Blood collection was performed 15–30 min after tDCS or sham stimulation. 

### 4.3. RNA-Seq on Blood Samples

Total RNA was isolated from peripheral blood with the Quick DNA/RNA miniprep plus kit (ZymoResearch, Irvine, CA, USA) according to the manufacturer’s instruction. The RNA purity was evaluated with NanoDrop Spectrophotometer 2000 (Thermo Fisher Scientific, Waltham, MA, USA) and RNA integrity number (RIN) was determined with Agilent 2100 Bioanalyzer (Agilent Technologies, Santa Clara, CA, USA).

Alpha and beta globin were depleted from total RNA with the GLOBINclear Kit (Invitrogen by Thermo Fisher Scientific), and sequin synthetic RNA spike-in controls were added to mRNA as sequencing internal controls [81]. Libraries were produced with QuantSeq 3′ mRNA-Seq Library Prep Kit for Ion Torrent (Lexogen GmbH, Vienna, Austria) according to the manufacturer’s instruction. The templates were prepared with the Ion PI Hi-Q OT2 200 Kit on the Ion OneTouch 2 System and sequenced on the Ion Proton System with Ion PI Hi-Q Sequencing 200 Kit (Ion Torrent by Thermo Fisher Scientific).

Demultiplexing and barcode trimming were performed automatically by the Torrent Suite Software (v.5.12.0) after each sequencing run, and data from multiple runs were merged in order to create a unique FASTQ file for each sample. Following the guidelines of the Lexogen manufacturer, we used cutadapt v1.14 [82] to cut poly-A tails and five bases from the 5′ prime end (which may contain mismatched bases eventually introduced by random priming). After trimming, all reads with a length below 30 bp and quality trimming below Q10 were discarded. The quality of processed data was evaluated analyzing the sequin spike-in transcripts using Kallisto [83] and the Anaquin package in R [81].

After QC, STAR aligner v. 2.6 [84] was used to compute reads alignment and gene counts based on the *Mus musculus* reference genome (mm10) and the ENCODE V19 comprehensive gene-set annotations. After merging data from all samples, only genes with more than 10 reads in at least 3 samples were included, resulting in a gene count matrix with 42 samples and 15,613 genes.

### 4.4. Whole Transcriptome Analysis

#### 4.4.1. Identification of Differentially Expressed Genes

The gene expression analysis was performed using the DESeq2 package in R, as described in [85]. Principal Component Analysis (PCA) was performed with prcomp package on DESeq2 log-transformed data in R [85]. Within the expression dataset, we identified 6 sample groups stratified by mouse strain, age and treatment as defined in Table 1. First, we analyzed the specific transcription profile of the 3xTg-AD mouse strain by comparing mice in the sham group according to the following contrasts: (i) three 3-month-old (3 mo.) 3xTg-AD mice vs. three 3 mo. WT mice; (ii) nine 6-month-old (6 mo.) 3xTg-AD mice vs. six 6 mo. WT mice; (iii) six 6 mo. vs. three 3 mo. WT mice (Figure 1). Differentially expressed genes (DEGs) were identified using the Likelihood Ratio Test (LRT) as implemented in DESeq2 comparing a reduced model including “RIN” and “site of stimulation” variables with a full model including RIN “site of stimulation” and group variables. “Site of stimulation” was included as covariate in the comparison of mice that underwent sham stimulation due to its strong correlation with the first principal component. We then investigated the effect of tDCS on transcription profiles of 6 mo. mice according to the following contrasts: (i) fourteen 3xTg-AD mice undergoing tDCS stimulation vs. nine 3xTg-AD mice undergoing sham stimulation; (ii) seven WT mice undergoing tDCS stimulation vs. six WT mice undergoing sham stimulation (Figure 1).

DEGs were identified through LRT, comparing a reduced model including RIN and site of stimulation variables and a full model including RIN, site of stimulation and stimulation variables. The DESeq2 independent filtering option was applied to maximize the number of genes that will have an adjusted *p*-value less than 0.1. False discovery rate BH FDR was used to control for multiple tests comparisons, and genes with FDR < 0.05 were considered as significantly modulated in subsequent analyses.

#### 4.4.2. Random Test Statistics on Venn Diagram

In order to test if the number of genes in the Venn intersections was higher than expected by chance, we applied a random test statistic. Briefly, on the overall set of 15,613 genes addressable in our analysis (background genes), we randomly sampled 3 groups of N genes, with N equal to the number of DEG in the three aforementioned comparisons (1st comparison N = 470; 2nd comparison N = 1551; 3rd comparison N = 271) and we counted the number of overlapping genes among the three groups. The procedure was repeated 10 million times. Empirical *p*-values were then calculated as the number of tests resulting in an equal or higher number of overlapping genes than those observed and reported in the Venn diagram of Figure 3.

#### 4.4.3. Over-Representation Analysis (ORA)

Gene-set enrichment analysis was performed for the significantly modulated genes (FDR < 0.05) using WebGestaltR v. 4.0.3 [86], the ORA enrichment method and considering the 15,613 genes analyzed as the background set. Enrichment categories considered were: GO Biological Process, GO Cellular Component, GO Molecular Function, KEGG and Reactome pathways. Only enrichment categories with more than 10 genes and less than 500 genes were analyzed, and those categories with FDR < 0.05 were considered significantly enriched. The affinity propagation method was used to reduce the number of gene sets.

## Figures and Tables

**Table 1:** Number of mice analyzed in this study stratified for strain, age, and site of stimulation.

Strain	Age	Stimulation	Number of Mice ^1^
3xTg-AD	3 months old	sham	3 (HP)
	6 months old	sham	9 (5 HP, 4 PFC)
		tDCS	14 (8 HP, 6 PFC)
C57BL/6	3 months old	sham	3 (HP)
	6 months old	sham	6 (HP)
		tDCS	7 (3 HP, 4 PFC)

^1^ Site of stimulation: HP: hippocampus; PFC: prefrontal cortex.

**Table 2:** List of top ten DEGs identified in the three comparisons.

Gene Name	Base Mean	log_2_ Fold Change	log_2_ Fold SE	*p* Value	FDR	Official Full Name
1st Comparison: 3 mo. 3xTg-AD vs. 3 mo. C57BL/6 mice						
*Npy*	3148	16.02	1.95	1.36 × 10^−33^	1.66 × 10^−29^	neuropeptide Y
*Gdpd3*	1025	13.24	1.87	2.48 × 10^−30^	1.52 × 10^−26^	glycerophosphodiester phosphodiesterase domain containing 3
*Dut*	552	−4.55	0.49	1.66 × 10^−23^	6.76 × 10^−20^	deoxyuridine triphosphatase
*Gm11942*	29,125	9.49	0.63	4.35 × 10^−23^	1.33 × 10^−19^	predicted gene 11,942 b22
*F2rl2*	711	−4.88	0.53	7.00 × 10^−23^	1.71 × 10^−19^	coagulation factor II (thrombin) receptor-like 2
*Klk1b22*	272	11.94	1.87	9.40 × 10^−23^	1.92 × 10^−19^	kallikrein 1-related peptidase
*Tpm4*	6746	−3.75	0.40	2.79 × 10^−22^	4.87 × 10^−19^	tropomyosin 4
*Serpine2*	798	−3.12	0.37	4.13 × 10^−19^	6.31 × 10^−16^	serine (or cysteine) peptidase inhibitor, clade E, member 2
*Acp1*	597	−3.65	0.44	3.29 × 10^−18^	4.47 × 10^−15^	acid phosphatase 1, soluble
*Josd2*	2113	4.65	0.49	3.91 × 10^−15^	4.78 × 10^−12^	Josephin domain containing 2
2nd Comparison: 6 mo. 3xTg-AD vs. 6 mo. C57BL/6 mice						
*Klk1b22*	770	12.65	0.90	9.89 × 10^−87^	1.42 × 10^−82^	kallikrein 1-related peptidase b22
*Gm11942*	35,739	8.60	0.35	3.51 × 10^−78^	2.53 × 10^−74^	predicted gene 11,942
*Aa465934*	665	4.77	0.25	1.05 × 10^−70^	5.03 × 10^−67^	lncRNA gene
*Bc018473*	297	11.30	0.91	8.18 × 10^−66^	2.94 × 10^−62^	cDNA sequence BC018473
*Gdpd3*	2066	9.60	0.48	3.58 × 10^−65^	1.03 × 10^−61^	glycerophosphodiester phosphodiesterase domain containing
*Cttnbp2*	326	5.89	0.39	1.91 × 10^−58^	4.57 × 10^−55^	cortactin Binding Protein 2
*Josd2*	5004	5.96	0.32	9.44 × 10^−57^	1.94 × 10^−53^	Josephin domain containing 2
*Fgfrl1*	85	−7.98	0.68	7.04 × 10^−44^	1.27 × 10^−40^	3fibroblast growth factor receptor-like 1
*Gm48905*	151	−7.56	0.57	1.60 × 10^−38^	2.55 × 10^−35^	predicted gene 48,905
*4930526A20rik*	129	5.30	0.39	1.56 × 10^−36^	2.24 × 10^−33^	pseudogene
3rd Comparison: 6 mo. C57BL/6 vs. 3 mo. C57BL/6 mice						
*Evpl*	47	9.50	1.32	1.03 × 10^−16^	1.45 × 10^−12^	envoplakin
*Slc13a2*	73	4.33	0.57	4.59 × 10^−14^	3.23 × 10^−10^	solute carrier family 13 (sodium-dependent dicarboxylate transporter), member 2
*Ddit4*	157	−2.96	0.44	4.77 × 10^−13^	2.24 × 10^−9^	DNA-damage-inducible transcript 4
*Folr1*	23	7.27	1.32	6.63 × 10^−11^	2.25 × 10^−7^	folate receptor 1 (adult)
*Acsm1*	32	8.71	1.45	7.98 × 10^−11^	2.25 × 10^−7^	acyl-CoA synthetase medium-chain family member 1
*Cyp4a12B*	109	6.82	1.08	5.43 × 10^−10^	1.28 × 10^−6^	cytochrome P450, family 4, subfamily a, polypeptide 12B
*Gm16136*	39	9.07	1.53	3.55 × 10^−9^	7.14 × 10^−6^	predicted gene 16,136
*B3gnt8*	936	2.19	0.34	4.18 × 10^−9^	7.36 × 10^−6^	UDP-GlcNAc:betaGal beta-1,3-N-acetylglucosaminyltransferase 8
*Ces2f*	16	7.40	1.42	9.79 × 10^−9^	1.53 × 10^−5^	carboxylesterase 2F
*Ddr1*	23	7.28	1.41	2.00 × 10^−8^	2.56 × 10^−5^	discoidin domain receptor family, member 1

**Table 3:** ORA results for the groups derived from Venn diagram.

**Pathways Enriched among Down-Regulated Genes of Group C**							
**Gene Set**	**Description**	**Size**	**Expect**	**Ratio**	***p*** **Value**	**FDR**	**Gene List**
**GO:0007596**	blood coagulation	117	13.99	64.33	1.1 × 10^−^^5^	0.034	*Apoe*, *F2rl2*, *Dmtn*, *Pros1*, *Serpine2*, *Pf4*, *Cd9*, *Hpse*, *Mpig6b*
**GO:0030193**	regulation of blood coagulation	50	0.60	10.04	2.7 × 10^−^^5^	0.045	*Apoe*, *Dmtn*, *Pros1*, *Serpine2*, *Cd9*, *Hpse*
**Pathways Enriched among Down-Regulated Genes of Group D**							
**Gene Set**	**Description**	**Size**	**Expect**	**Ratio**	***p*** **Value**	**FDR**	**Gene List**
**GO:0051591**	response to cAMP	66	0.11	35.96	4.0 × 10^−^^6^	0.0330	*Dusp1*, *Fos*, *Fosb*, *Junb*
**Pathways Enriched among Down-Regulated Genes of Group A**							
**Gene Set**	**Description**	**Size**	**Expect**	**Ratio**	***p*** **Value**	**FDR**	**Gene List**
**R-MMU-449147**	Signaling by Interleukins	247	3.01	4.65	01.9 × 10^−^^6^	0.008	*Lck*, *Il17ra*, *Psmb1*, *Peli1*, *Vamp2*, *Stat5b*, *Psmb8*, *Il1r2*, *Il1b*, *Nfkbib*, *Il15*, *Pik3cb*, *Osm*, *Mapk1*
**Pathways Enriched Among Up-Regulated Genes of Group B**							
**Gene Set**	**Description**	**Size**	**Expect**	**Ratio**	***p*** **Value**	**FDR**	**Gene List**
**GO:0061912**	selective autophagy	42	2.03	4.93	2.3 × 10^−^^5^	0.042	*Sqstm1*, *Cdc37*, *Sptlc1*, *Prkn*, *Rb1cc1*, *Optn*, *Becn1*, *Wdr81*, *Mapk3*, *Clec16a*
**GO:0010466**	negative regulation of peptidase activity	161	7.78	2.70	3.1 × 10^−^^5^	0.043	*Bcl2l1*, *Birc5*, *Arrb1*, *Rffl*, *Cast*, *Pak2*, *Birc6*, *Crim1*, *C3*, *Dnajb6*, *Ints1*, *Rps6ka3*, *Ngp*, *Ltf*, *Dhcr24*, *Gpi1*, *Fcmr*, *Usp14*, *Wfdc21*, *Gpx1*, *Wfdc3*
**Pathways Enriched among DEGs of Group F**							
**Gene Set**	**Description**	**Size**	**Expect**	**Ratio**	***p*** **Value**	**FDR**	**Gene List**
**GO:0030216**	keratinocyte differentiation	102	1.32	8.3	7.7 × 10^−^^8^	0.0006	*Grhl1*, *Cers3*, *Ptgs2*, *Evpl*, *Kdf1*, *Lats1*, *Lce1l*, *Dsp*, *Lce1a1*, *Cyp26b1*, *Lce1a2*
**GO:0030036**	actin cytoskeleton organization	485	6.27	3.0	1.6 × 10^−^^5^	0.033	*Scin*, *Tnfaip1*, *Myo1b*, *Cobl*, *Dpysl3*, *Epb41l5*, *Epb41l4b*, *Fgr*, *Dlc1*, *Sorbs2*, *Cgnl1*, *Kif23*, *Kank1*, *Arhgef5*, *Rhov*, *Prr5*, *Lats1*, *Tacstd2*, *Gm14137*
**R-MMU-211945**	Phase I—Functionalization of compounds	73	0.94	7.4	4.2 × 10^−^^5^	0.048	*Fdxr*, *Aldh3a1*, *Aldh1a7*, *Adh7*, *Ces2f*, *Cyp26b1*, *Cyp4a12b*
**GO:0050891**	multicellular organismal water homeostasis	32	0.41	12.1	5.1 × 10^−^^5^	0.048	*Grhl1*, *Tfap2b*, *Aqp3*, *Kdf1*, *Cyp26b1*
**GO:0045104**	intermediate filament cytoskeleton organization	34	0.44	11.4	6.9 × 10^−^^5^	0.048	*Krt18*, *Dst*, *Pkp1*, *Evpl*, *Dsp*

**Table 4:** List of 25 genes significantly modulated by tDCS in 3xTg-AD mice.

Genes	Base Mean	log_2_ Fold Change	log_2_ Fold SE	*p* Value	FDR	Official Full Name
*Pabpc1*	363,466	−1.17	0.25	2.0 × 10^−6^	0.010	poly(A) binding protein, cytoplasmic 1
***Crat***	**1846**	**−0.86**	**0.20**	**1.4 × 10^−5^**	**0.022**	**carnitine acetyltransferase**
*Casp4*	113	1.33	0.28	1.1 × 10^−5^	0.022	caspase 4, apoptosis-related cysteine peptidase
*Il13ra1*	181	1.15	0.27	3.6 × 10^−5^	0.042	interleukin 13 receptor, alpha 1
*Septin1*	928	−0.81	0.21	8.3 × 10^−5^	0.049	septin 1
*Ccr1*	262	1.33	0.31	5.9 × 10^−5^	0.049	chemokine (C-C motif) receptor 1
***Ybx3***	**48,855**	**−0.79**	**0.21**	**7.7 × 10^−5^**	**0.049**	**Y box protein 3**
*Ptp4a3*	35,887	−0.75	0.19	8.1 × 10^−5^	0.049	protein tyrosine phosphatase 4a3
*Ap1b1*	458	−0.65	0.17	1.1 × 10^−4^	0.049	adaptor protein complex AP-1, beta 1 subunit
*Tmpo*	362	0.57	0.14	1.2 × 10^−4^	0.049	thymopoietin
*Fcgr3*	583	0.96	0.24	1.1 × 10^−4^	0.049	Fc receptor, IgG, low affinity III
*Stfa2l1*	521	1.35	0.33	1.3 × 10^−4^	0.049	stefin A2
*Cd52*	6685	0.53	0.14	2.6 × 10^−4^	0.049	CD52 antigen
*Cbfa2t3*	366	−0.72	0.20	2.6 × 10^−4^	0.049	CBFA2/RUNX1 translocation partner 3
*Ogdh*	1902	−0.70	0.19	2.3 × 10^−4^	0.049	oxoglutarate (alpha-ketoglutarate) dehydrogenase (lipoamide)
*Retnlg*	9116	1.36	0.34	1.6 × 10^−4^	0.049	resistin-like gamma
*Il1r2*	262	2.04	0.52	2.6 × 10^−4^	0.049	interleukin 1 receptor, type II
*Vim*	948	0.81	0.21	2.2 × 10^−4^	0.049	vimentin
*Tlr13*	263	0.93	0.24	2.4 × 10^−4^	0.049	toll-like receptor 13
***Ago2***	**6349**	**−0.67**	**0.18**	**1.9** **× 10** **^−4^**	**0.049**	**argonaute RISC catalytic subunit 2**
*Rnf123*	285	−0.94	0.26	1.9 × 10^−4^	0.049	ring finger protein 123
*Mical3*	269	−0.85	0.23	2.0 × 10^−4^	0.049	microtubule associated monooxygenase, calponin and LIM domain containing 3
*Cstdc4*	472	1.40	0.35	1.7 × 10^−4^	0.049	predicted gene 5483
*Sirpb1b*	133	1.19	0.31	1.8 × 10^−4^	0.049	signal-regulatory protein beta 1B
*Mrpl33*	1057	0.85	0.22	2.0 × 10^−4^	0.049	mitochondrial ribosomal protein L33

The three genes belonging to Group C are reported in bold.

## Data Availability

The data discussed in this publication have been deposited in NCBI’s Gene Expression Omnibus [87] and they will be accessible through GEO Series accession number GSE178662 (https://www.ncbi.nlm.nih.gov/geo/query/acc.cgi?acc=GSE178662) (accessed on 19 July 2021).

## Supplementary Materials

The following are available online at https://www.mdpi.com/article/10.3390/ijms22147629/s1, Supplementary Figures: This file includes two supplementary figures. Supplementary Figure S1 depicts the scatterplot of the correlation between the expected and observed sequins concentration. Supplementary Figure S2 is a scree plot of the percentage of variance explained by the first ten principal components. Supplementary Tables: This excel file includes two supplementary tables. Supplementary Table S1 reports the summary statistics derived from the LRT analysis performed with DESeq2 software for the 15,613 analyzed genes. This table reports the results of the three comparisons performed among sham mice and the two comparisons between mice undergoing sham or tDCS stimulation. In the table, there are three additional columns: “Venn-intersection Groups”, “KEGG Pathway: Alzheimer disease” and “Ochi’s study DEGs”. “Venn-intersection Groups” indicates the group of the Venn intersection; “KEGG Pathway: Alzheimer disease” indicates genes belonging to this pathway; “Ochi’s study DEGs” indicates genes that were DEGs in the Ochi study [15]. Supplementary Table S2 summarizes the results of the ORA analyses for the three comparisons reported in the text.
