# Supplementary material for: Whole Blood Transcriptome Characterization of 3xTg-AD Mouse and Its Modulation by Transcranial Direct Current Stimulation (tDCS)
Source: Int J Mol Sci. 2021 Jul 16;22(14):7629. doi: 10.3390/ijms22147629 (PMC8306644; doi:10.3390/ijms22147629)

Supplementary Figures

Supplementary Figure S1. Scatterplot of the of the correlation between the expected and observed sequins' concentration.

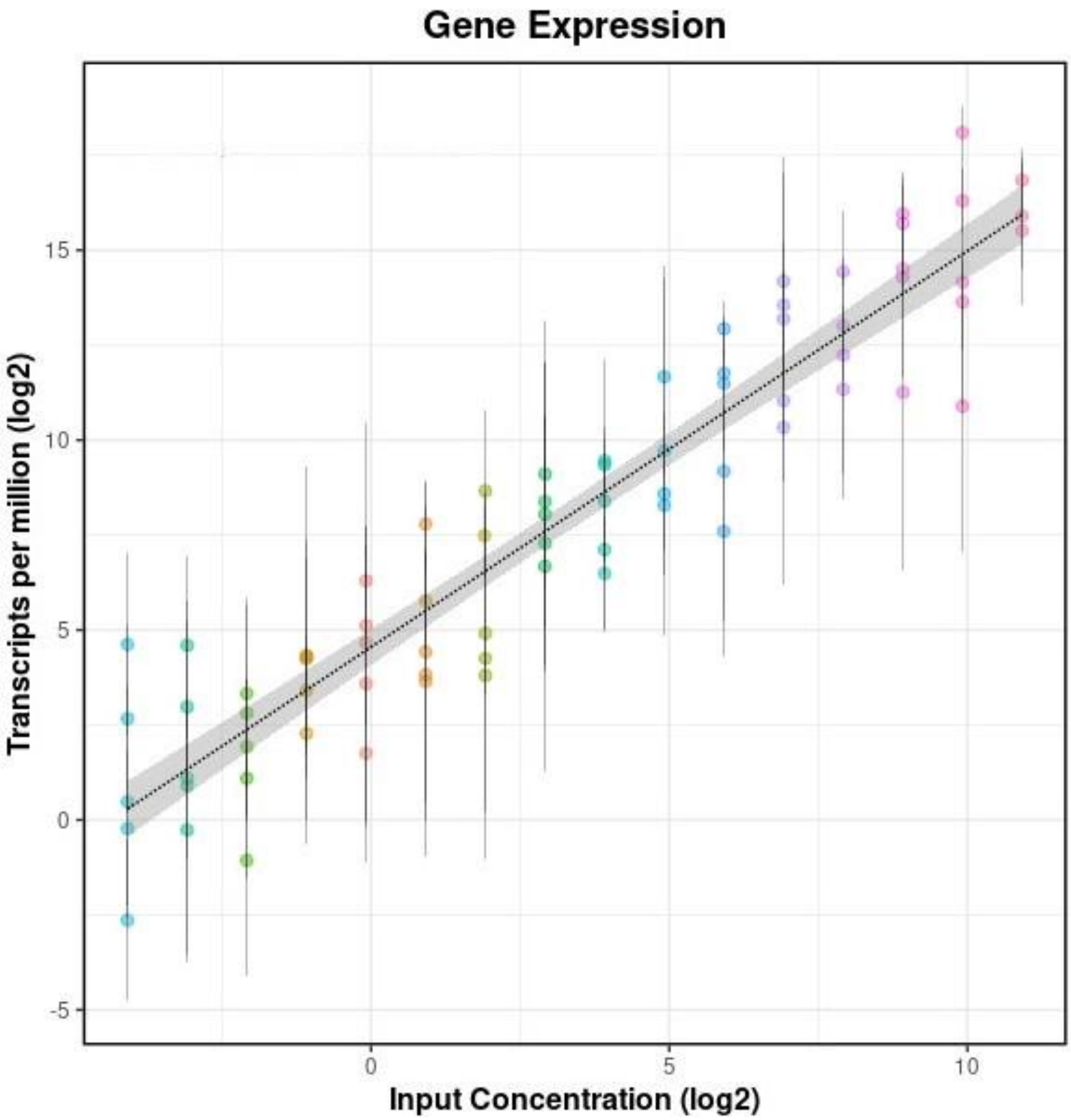

Supplementary Figure S2. Scree plot of the percentage of variance explained by the first ten principal components.

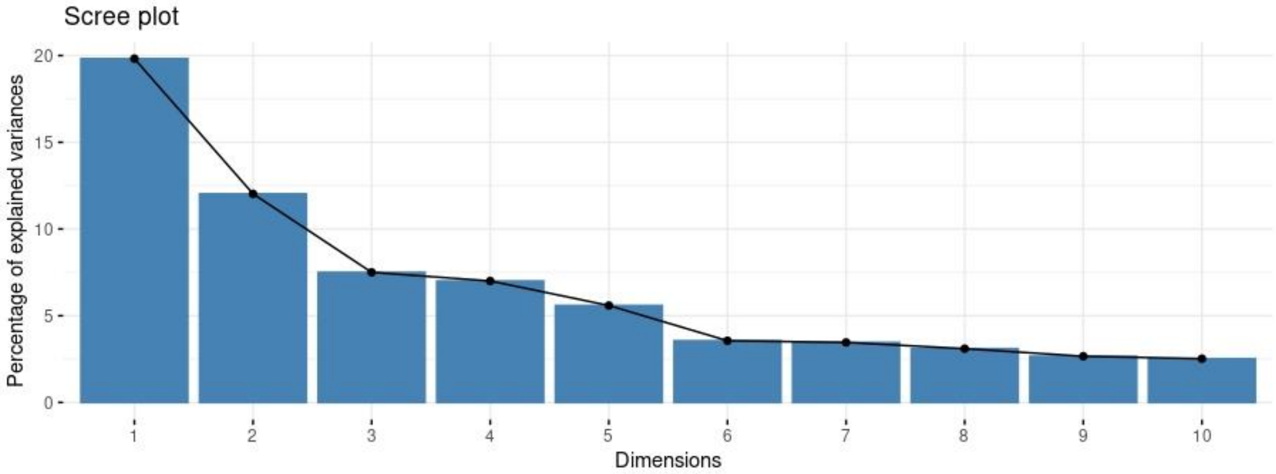

Supplement: Supplementary file 1 [file ijms-22-07629-s001.zip › ijms-1279524_Supplementary_Figures.pdf]
